# Does short inter-pregnancy interval predicts the risk of preterm birth in Northern Ethiopia?

**DOI:** 10.1186/s13104-019-4439-1

**Published:** 2019-07-15

**Authors:** Merhawi Brhane, Brhane Hagos, Mulugeta Woldu Abrha, Haftom Gebrehiwot Weldearegay

**Affiliations:** 1 College of Health Sciences, Mekelle University, Mekelle, Ethiopia; 2 College of Health Sciences, Dilla University, Dilla, Ethiopia; 3 Tigray Health Research Institute, Mekelle, Ethiopia

**Keywords:** Inter pregnancy interval, Incidence, Preterm birth, Cohort study, Northern Ethiopia

## Abstract

**Objective:**

The study aimed to assess the effect of inter pregnancy interval on preterm birth in Northern Ethiopia: prospective cohort study.

**Result:**

This study showed that, total incidence of premature birth was 10.4%. Among mothers with short inter pregnancy interval the incidence of preterm birth was 39 (25.9%).Whereas, among mothers who had recommended inter pregnancy interval was 9 (2.9%). Short inter-pregnancy interval [adjusted hazard ratio (AHR): 6.85, 95% confidence interval (CI) 3.07–15.31], antenatal care (ANC) visit 1–3 times (AHR: 2.24, 95% CI 1.04–4.85), complication during pregnancy (AHR: 3.16, 95% CI 1.58–6.33) and birth defect (AHR: 8.01, 95% CI 2.56–25.07) were predictors of premature birth.

## Introduction

Preterm birth (PTB) is defined as a delivery which occurs at less than 37 completed weeks of gestation. It is classified as extremely preterm (< 28 weeks), very preterm (28 to < 32 weeks) and moderate to late preterm (32 to < 37 weeks) [1]. Globally, 15 million babies are born too early every year, which are more than 1 in 10 babies. Approximately 1 million children die each year due to complications of preterm birth. Moreover, many survivors face a lifetime of disability, learning incapacities, visual and hearing problems [2].

Systemic review done in 2007 showed that, 9.6% of all births were preterm, which translates to about 12.9 million births definable as preterm. Approximately 85% of this burden was concentrated in Africa and Asia, where 10.9 million births were preterm [3]. Based on UNICEF report among the neonates delivered in Ethiopia 10% of them are preterm [4]. Different studies conducted in Ethiopia revealed that the incidence of preterm birth ranges from 4.4 to 25.9% [5–9]. And it also accounts 28% of newborn deaths in Ethiopia [10].

Other studies indicates that history of abortion, still birth, preterm birth, Premature rupture of fetal membrane, history of bleeding during the pregnancy, pregnancy induced hypertension, HIV infection, substance intake, history of low birth weight and short inter-pregnancy interval (IPI) were the factors leading to preterm birth [4–8].

Furthermore, women with short interval between pregnancies are at increased risk of preterm birth. However, whether this association is confounded by other risk factors, including various aspects of socioeconomic status, ethnicity, demographics, and lifestyle is unclear [11]. Therefore this study aimed to assess the effect of inter-pregnancy interval and preterm birth in Ethiopia. This will help health providers to have adequate knowledge and intervene early in the antenatal care visit and also might have an input to programmers and policy makers.

## Main text

### Methods and material

#### Study setting, design and sample size

Health facility based prospective cohort study design was conducted in Tigray Region, Northern Ethiopia, located at distance of 1087 km from Addis Ababa, the capital city of Ethiopia. Source population was child bearing mothers who are attending delivery in north western zone health facilities of Tigray.

The sample size of 480 pregnant women was calculated using Epi-info-Calc. statistical software with the assumption of 95% CI, power of 90%, ratio of unexposed to exposed was 2:1 and percentage of exposed among preterm birth of 8.5% (14), odds ratio of 2.7, Loss to follow-up rate was estimated to be 10%.

Four hospitals and eight health centers were selected randomly from North West zone health facilities. Study participants were proportionally allocated to each health facilities based on eligible pregnant mothers for ANC. Lastly, participants were included through systematic sampling method until the desired sample achieved.

#### Data collection procedure and quality assurance

Data was collected using standardized, structured and face to face interviewer questionnaire and card review. Data quality was managed by trained three Bachelors degree of science holders fluent in the local language (Tigrigna). Pre-test, daily supervision, spot checking and reviewing completed questionnaire was conducted.

#### Explanatory measurements

Gestational age: was estimated as the interval in completed weeks from the last normal menstrual period (LNMP) to the child’s date of birth. When there are extra days it is counted to the near lowest gestational age but if the mother didn’t remember or recall her LNMP; gestational age was estimated from ultra sound result through reviewing the mothers chart.

Inter-pregnancy interval: was documented as the time interval between delivery of the first pregnancy and the conception of the subsequent pregnancy.

Short inter-pregnancy interval: mothers with inter-pregnancy interval from 0 to 24 months.

Recommended inter-pregnancy interval: mothers with inter-pregnancy interval from 24 to 36 months or longer.

#### Outcome measures

Censor: mothers with normal weight, low birth weight, still birth, and congenital anomaly.

Event: preterm birth.

#### Statistical analysis

Data was entered into Epi Data 3.5.1 statistical software and analyzed using SPSS version 20.0™ package. The results were presented in tables and texts using descriptive statistics such as mean, standard deviation, frequency and percentages.

The relationship between the event and explanatory variables was analyzed using Cox bivariate proportional regression model. Before fitting the covariate into the model proportional hazard assumption was checked using Log (−Log) S (t) plots.

In order to identify independent predictors a multivariate Cox-proportional adjusted model was fitted with those variables p-value ≤ 0.25 in bivariate cox proportional regression analysis. Crude and adjusted hazard ratios with their 95% Confidence interval (CI) were estimated and p-value less than 0.05 were used to declare the presence of significant association.

### Results

#### Socio demographic characteristics of participants

Four hundred eighty pregnant women were followed with 20 (4.17%) lost in follow-up until delivery. The mean age of mothers was 30 (SD ± 5.92) ranging from 15 to a maximum of 48 years and above half 260 (56.5%) were between 25 and 34 years. Almost all or 450(97.8%) of the participants were married. Two-fifth 164 (35.7%) of the study participants had no formal education. About maternal occupation four out of ten participants 205(44.6%) were housewife and above half 273 (59.3%) of the participants come from Urban (Table 1).

**Table 1:** Socio-demographic characteristic of pregnant mothers in Tigray, Northern Ethiopia, 2018 (N = 460)

Categories	Frequency	Percentage
Age in completed years		
15–24	75	16.3
25–34	260	56.5
35–48	125	27.2
Marital status		
Married	450	97.8
Unmarried	4	0.9
Divorced/widowed	6	1.3
Educational level		
None	164	35.7
Primary	82	17.8
Secondary	135	29.3
College and above	79	17.2
Occupation		
House wife	205	44.6
Farmer	125	27.2
Employed	130	28.3
Residence		
Rural	187	40.7
Urban	273	59.3

#### Past-obstetrics and current pregnancy characteristics

Among the cohort women two-third 308 (67.0%) of them had recommended inter pregnancy interval and three-fourth 346 (75.2%) of them had ≤ 4 pregnancy with two-third 312 (67.8%) of them were multiparous. One out of ten (n = 418) women experience perinatal death in their preceding pregnancy. Around three-fourth 332 (72.2%) of the participants had planned pregnancy. Majority 445 (96.7%) of the pregnant women had at least one ANC checkup and four hundred thirteen (89.8%) of the pregnant women had maternal obstetrical complication during their current pregnancy. Eight out of ten (n = 375) of the study participants had hemoglobin level above 11 g/dl and eighty percent (n = 374) participants had delivered through spontaneous vaginal delivery for their current baby with a majority, 281 (61.1%) of them gave male baby by sex (Table 2).

**Table 2:** Past-obstetrics and current pregnancy characteristics of pregnant mothers in Tigray, Northern Ethiopia, 2018 (N = 460)

Category	Frequency	Percentage
Inter pregnancy interval		
Recommended	308	67.0
Short	152	33.0
Gravidity		
≤ 4 pregnancy	346	75.2
≥ 5 pregnancy	114	24.8
Parity		
Primipara	148	32.2
Multipara	312	67.8
History of perinatal death		
No	418	90.9
Yes	42	9.1
Planned pregnancy		
No	128	27.8
Yes	332	72.2
ANC follow up		
No	15	3.3
Yes	445	96.7
Number of ANC visits		
≥ 4 times	225	57.3
1–3 times	190	42.7
Initiation of ANC visit		
Within 16 weeks	22	4.9
24–28 weeks	244	54.8
28–32 weeks	169	38.0
34–36 weeks	10	2.2
Current pregnancy maternal complication^a^		
No	413	89.8
Yes	47	10.2
Maternal hemoglobin level (g/dl)		
≥11	375	81.5
<11	85	18.5
Maternal Rh factor		
Positive	449	97.6
Negative	11	2.4
Maternal and fetal intra partum complication^b^		
No	414	90.0
Yes	46	10.0
Mode of delivery		
Cesarean section	45	9.8
Instrumental	18	3.9
Induction/augmentation	23	5.0
Spontaneous vaginal delivery	374	81.3
Sex of newborn		
Male	281	61.1
Female	179	38.9

^a^Current pregnancy maternal problem: APH, preeclampsia, anemia, uterine rupture/scar dehiscence

^b^Maternal and fetal Intra partum complication: PPH, obstructed labor, prolonged labor, uterine rupture/scar dehiscence

#### Incidence and predictors of premature birth

The total incidence of premature birth in this study was 10.4%. Incidence among mothers exposed for short inter pregnancy interval was 39 (25.9%) and this indicates that around 3 out of 10 mothers who are exposed for short inter pregnancy interval have premature baby. But the incidence among mothers with recommended inter-pregnancy interval was 9 (2.9%).

The predictors for premature birth were mothers with short inter-pregnancy interval had around seven times (AHR: 6.85, 95% CI 3.07–15.31) hazard of bearing premature birth than those with recommended inter-pregnancy interval; also mothers with 1-3 times ANC visit had two times (AHR: 2.24, 95% CI 1.04–4.85) likelihood to have premature birth than those who have greater than or equal to 4 visits and pregnant mother who had any problem during her current pregnancy had 3 times (AHR: 3.16, 95% CI 1.58–6.33) more hazard to give premature birth than their counter parts. And those pregnant who gave birth a newborn with birth defect was eight times (AHR: 8.01, 95% CI 2.56–25.07) more hazardous than those with normal birth. However, maternal residence, planned pregnancy, history of perinatal death and maternal hemoglobin level were significant at bi-variable but in significant in multivariable regression analysis (Table 3).

**Table 3:** Bi-variable and multivariable cox-regression analysis for predictors of premature birth, Northern Ethiopia, 2018 (N = 460)

Characteristics	N	Outcome n (%)		Hazard ratio, 95% CI	
		Event	Censored	Crude	Adjusted
IPI					
Short	152	39 (25.9)	113 (74.1)	9.5 (4.6–19.6)	*6.85 (3.07–15.31)*
Recommended	308	9 (2.9)	299 (97.1)	1	
Residence					
Rural	187	30 (16)	157 (84)	2.5 (1.37–4.41)	1.2 (0.61–2.35)
Urban	273	18 (6.6)	255 (93.4)	1	
Planned pregnancy					
No	128	22 (17.2)	106 (82.8)	2.26 (1.28–3.99)	1.11 (0.57–2.13)
Yes	332	26 (7.8)	306 (92.2)	1	
Number of ANC visits					
≥ 4	255	11 (4.3)	244 (95.7)	1	
1–3	190	31 (16.3)	159 (83.7)	4.0 (2.01–7.97)	*2.24 (1.04–4.85)*
Complication in current pregnancy					
No	413	33 (8.0)	380 (92)	1	
Yes	47	15 (31.9)	32 (68.1)	4.78 (2.59–8.79)	*3.16 (1.58–6.33)*
History of perinatal death					
No	418	40 (9.6)	378 (91.4)	2.16 (1.01–4.61)	0.85 (0.36–2.03)
Yes	42	8 (19)	34 (81)	1	
Newborn birth defect					
No	452	43 (9.5)	409 (90.5)	1	
Yes	8	5 (62.5)	3 (37.5)	12.97 (5.11–32.87)	*8.01 (2.56–25.07)*
Mother hemoglobin level (g/dl)					
≥ 11	375	32 (8.5)	343 (91.5)	2.33 (1.28–4.25)	0.91 (0.46–1.79)
< 11	85	16 (18.8)	69 (81.2)	1	
Maternal age (years)					
15–24	75	9 (12)	66 (88)	1.68 (0.67–4.23)	
25–34	260	30 (11.5)	230 (88.5)	1.64 (0.78–3.46)	
35–48	125	9 (7.2)	116 (92.8)	1	

### Discussion

This study showed the incidence of spontaneous premature birth was decreasing from the previous studies done in Ethiopia. Short inter-pregnancy interval, number of ANC visit, newborn birth defect and presence of complication during pregnancy were the predictors of preterm birth.

The finding of this study revealed that incidence of premature birth was 10.4% which is almost similar with the study done in Amhara Regional state, Ethiopia in which the preterm birth was 11.6% [8]. However, this result was higher than the study done in Mexico (7.4%) [11] and Southern India (5.4%) [12]. This inconsistency of the finding might be due to the socioeconomic difference and awareness on family planning use. And this result was again lower than the studies done in different parts of Ethiopia such as Addis Ababa (16.15%), Gondar (14.3%) and Jimma (25.9%) [5–7]. This dissimilarity could be due to the success of different programs introduced by federal Ministry of Health to improve health service quality delivered to pregnant women including pre-pregnancy and pregnancy health care in the last decades.

Other result indicated that, short inter-pregnancy interval increases the occurrence of premature birth, which is similar with the study done in Ethiopia [7], Pakistan [13]. The result from Pakistan indicated that mothers with inter-pregnancy interval less than a year were at a higher frequency of preterm birth as compared to mothers with recommended inter-pregnancy interval. The reason could be short inter-pregnancy interval mothers cannot recover from the biological stresses imposed by the preceding pregnancy resulting in diminution of macronutrient supplementation in maternal body, folate depletion, cervical insufficiency, vertical transmission of infections, incomplete healing of uterine scar and abnormal remodeling of endometrial blood vessels, anemia and increasing the risks of certain other factors achieving pregnancy outcomes [14–16]. So promoting and encouraging the recommended inter-pregnancy interval among couples will reduce the incidence of preterm birth.

In addition, number of ANC visit were another predictor of incidence of preterm birth which was also determined in the studies done in Debremarkos and Gondar Ethiopia [6, 8], Ghana **[**17] and China [18]. This could be due to the importance of recommended ANC follow up and proper visit on detection and early treatment of the problem or any complications. When pregnant mothers come to health facility with any problem or complication that can lead to preterm birth, she might get early treatment so as to prevent the occurrences of preterm birth and other related adverse obstetric outcomes. Thus, mothers should have to follow the recommended ANC visits.

The other predictor in this study was presence of complication during pregnancy (APH, preeclampsia and anemia) which is similar with studies done in Ethiopia [5–8], Ghana [14], China [15], Mexico [11] and southern India [12]. Explicitly, hypertension during pregnancy negatively impacts placental blood flow and leads to poor fetal growth and obstetric emergencies, which increase the risk of having a preterm birth. Therefore early identification, detection and management are crucial for pregnant mothers during their recommended ANC visits.

Having birth defect was the other predictor of preterm birth which is comparable with the study done in Ethiopia [7], New-York United States [19]. The causes of most birth defects and the reasons why these birth defects might contribute to being preterm birth remain unknown. While it is likely that the most common of these defects result from the interface of genetic and environmental risk factors, the identification of specific modifiable risk factors continues to be an important research and public health priority. The possible pathways that could explain the association between preterm birth and birth defects were have a shared risk factors (e.g., maternal smoking, obesity) to cause both birth defects and preterm birth. On the other side, a particular risk factor (e.g., valproic acid, insufficient peri-conceptional folic acid) could lead to a spina bifida, and the presence of that birth defect could then be part of the underlying mechanism causing in a preterm delivery [20]. This suggests that clinical interventions aimed at preventing birth defects may have added benefits in averting the incidences of preterm birth and low birth weight.

### Conclusion

This study indicates that incidence of premature birth is decreasing when compared with studies done in other parts of Ethiopia. Our findings, in conjunction with those of other studies, strongly suggest that a short inter pregnancy interval is a causal factor for preterm birth. Number of ANC visit, complication during pregnancy and being birth defect are also the predictors for premature birth.

Therefore, the evaluation of the outcomes of primary prevention programs based on this putative causal factor would resolve the matter and health professionals should target to improve maternal education and counseling on recommended ANC visits and the adverse effect of shorter IPIs on subsequent birth, better access to contraception to increase intervals between births and therefore reduce the risk of preterm birth.

Enhancing social marketing approaches emphasized the need to seek early and focused antenatal care to those non-booked mothers is also mandatory. Better management of obstetric complications and research to elucidate the mechanisms by which they cause preterm birth, offers a practical approach of reducing the high preterm birth rates.

## Limitation of the study

There are some limitations on this study. There was lost to follow-up of participants and it was not possible to enroll mothers who had difficult to estimate their gestational age that is unknown last normal menstrual period (LNMP) and missed the ultra sound result in her chart.

## Data Availability

The datasets used and/or analyzed during the current study is available from the corresponding author on request.

## Abbreviations

ANC: antenatal care
IPI: inter pregnancy interval
PTB: preterm birth
